# Current research status of risk assessment models for oral squamous cell carcinoma based on microbiome databases

**DOI:** 10.3389/fmicb.2026.1894946

**Published:** 2026-10-26

**Authors:** Guan-Lin Li, Zhu-Chao Zhang, Xiao-Ming Lyu, Mo-Xi Li, Pei-Yi Lin, Hui-Shan Yu, Yi-Yang Zhu, Guo-Hao Zhang, Cheng-Gang Duan, Yoo Cheung, Wei Li, Zhi-Jian Zhang, Tian-Song Xu, Feng Chen, Ming-Wei Huang

**Affiliations:** 1 Department of Oral and Maxillofacial Surgery, Peking University School and Hospital of Stomatology and National Center for Stomatology and National Clinical Research Center for Oral Diseases and National Engineering Research Center of Oral Biomaterials and Digital Medical Devices, Beijing, China; 2 Department of Oral and Maxillofacial Surgery, Guiyang Stomatological Hospital, Guiyang, Guizhou, China; 3 Fifth Clinical Division, Peking University School and Hospital of Stomatology and National Center for Stomatology and National Clinical Research Center for Oral Diseases and National Engineering Research Center of Oral Biomaterials and Digital Medical Devices, Beijing, China; 4 Lingchuan Stomatology Inc., Beijing, China; 5 Department of Respiratory and Critical Care Medicine, The Second Medical Center and National Clinical Research Center for Geriatric Disease, Chinese PLA General Hospital, Beijing, China; 6 Central Laboratory, Peking University School and Hospital of Stomatology and National Center for Stomatology and National Clinical Research Center for Oral Diseases and National Engineering Research Center of Oral Biomaterials and Digital Medical Devices, Beijing, China

**Keywords:** 16S rRNA sequencing, biomarker, machine learning, microbiome database, oral microbiome, risk prediction

## Abstract

Oral squamous cell carcinoma (OSCC) is one of the most common oral malignancies. Due to insidious early symptoms, high invasiveness, and frequent cervical lymph node metastasis, patient survival remains poor. Recent studies demonstrate significant associations between oral dysbiosis and OSCC. To address limited early screening and suboptimal survival rates, this review systematically summarizes OSCC microbiome research and disease risk assessment applications. Studies reveal marked oral dysbiosis in patients with OSCC, characterized by enrichment of *Fusobacterium* and *Porphyromonas*, reduced *Neisseria* abundance, and altered community stability. These promote tumorigenesis through chronic pro-inflammatory signaling, the production of carcinogenic metabolites, and direct oncogenic pathway interference. Consequently, the oral microbiome offers non-invasive, sensitive biomarkers for early detection. We identify and distinguish four categories of predictive models in the current literature: (1) detection/discrimination of prevalent OSCC, (2) prediction of malignant transformation from oral potentially malignant disorders, (3) prediction of recurrence or metastasis after treatment, and (4) prognostic models for survival prediction. Existing databases include comprehensive and OSCC-specific collections with multi-omics integration. Risk assessment models encompass traditional statistics, machine learning, deep learning, and multimodal fusion, and have shown promising performance in retrospective cohorts. However, standardized databases are lacking, and existing models face confounding interference, limited generalizability, and insufficient interpretability. Furthermore, current predictive models are predominantly based on 16S rRNA sequencing (bacteriome-centric), whereas comprehensive risk assessment requires integrating interkingdom entities, including fungi, viruses, and archaea, through metagenomic and virome profiling. Critically, the distinction between promising retrospective discrimination and validated prospective clinical utility remains a major translational barrier. Future directions include developing standardized longitudinal databases, integrating multi-omics, using explainable artificial intelligence, and conducting prospective validation studies to achieve personalized precision treatment.

## 1 Introduction

Oral squamous cell carcinoma (OSCC) represents the most prevalent head and neck malignancy, comprising over 90% of all oral cancer diagnoses. It typically manifests in anatomical sites such as the tongue, floor of the mouth, buccal mucosa, hard palate, and retromolar trigone (Dhingra, 2025). According to GLOBOCAN 2022, lip and oral cavity cancers accounted for approximately 389,485 new cases and 188,230 deaths globally (Bray et al., 2024). Notwithstanding significant diagnostic and therapeutic advancements, the 5-year survival rate remains stagnant at approximately 50%, primarily because approximately 60% of patients are diagnosed at advanced stages (Dhingra, 2025). Regions such as Southeast Asia and Oceania exhibit a particularly high disease burden, driven by lifestyle factors, such as betel nut chewing, tobacco use, and excessive alcohol consumption (Hernández-Morales et al., 2023).

Clinically, early-stage OSCC symptoms are often subtle and frequently misidentified as benign lesions. Coupled with high invasiveness and a propensity for cervical lymph node metastasis, this delay in diagnosis leads to significant risks of postoperative recurrence and functional morbidity (Intini et al., 2025). Traditional screening modalities, which rely on visual inspection and invasive biopsy, are often impractical for large-scale population screening or continuous dynamic monitoring (Pierfelice et al., 2024). Consequently, there is a critical need to develop sensitive, non-invasive tools for early detection and precise risk stratification (Figure 1).

**Figure 1:**
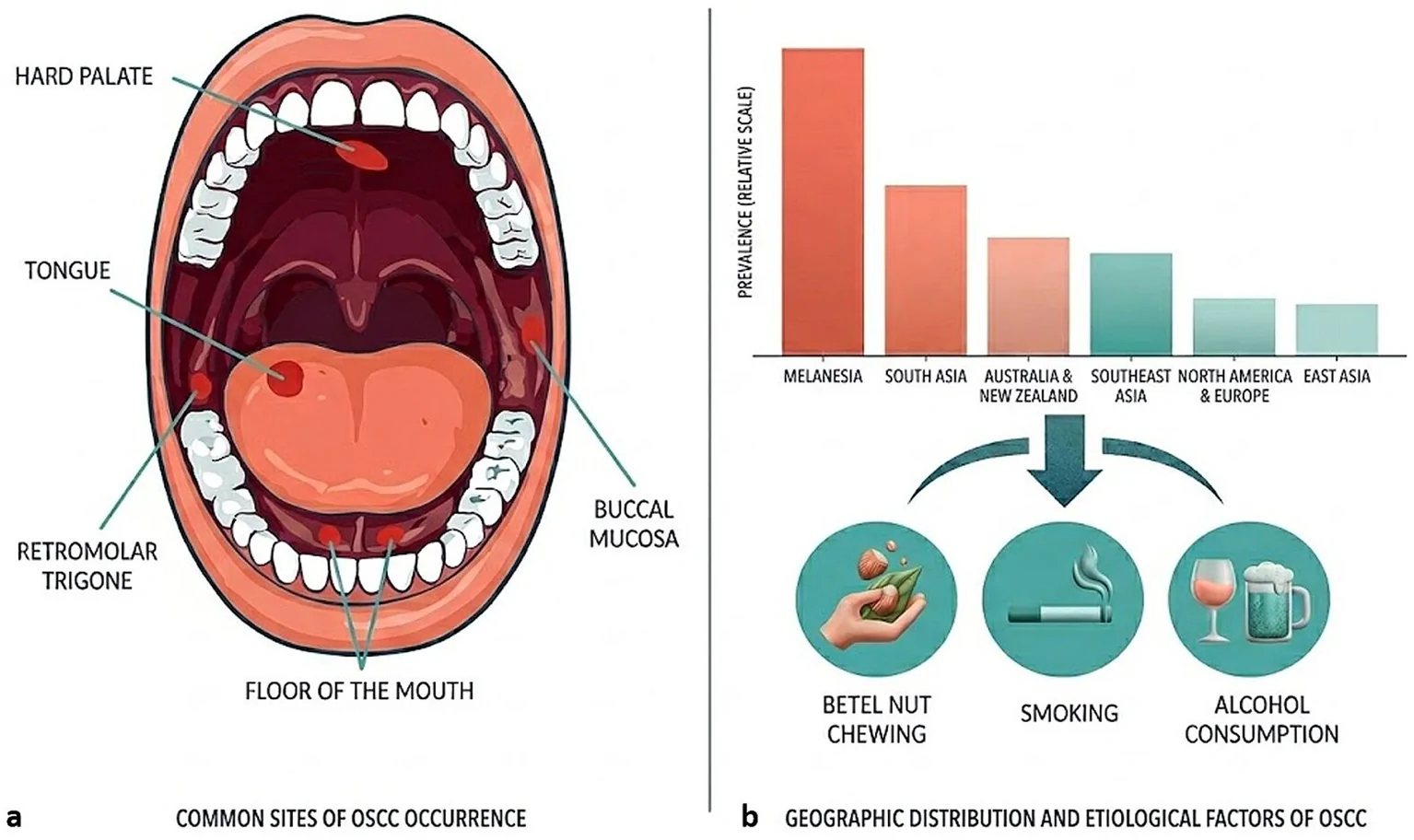
Clinical characteristics and epidemiology of oral squamous cell carcinoma (OSCC). **(a)** Common anatomical sites of OSCC occurrence: highlighting the hard palate, tongue, retromolar trigone, floor of the mouth, and buccal mucosa. **(b)** Geographic distribution and etiological factors: The bar chart illustrates the relative prevalence across different global regions (highest in Melanesia and South Asia), while the icons identify betel nut chewing, smoking, and alcohol consumption as the primary lifestyle risk factors.

In recent years, the oral microbiome has emerged as a pivotal environmental determinant of OSCC pathogenesis. As the second-largest microbial reservoir in the human body, the oral cavity hosts a complex community of over 700 species (Sukmana et al., 2024). Research indicates that the homeostatic symbiotic state in OSCC patients shifts toward significant dysbiosis (Wang et al., 2025a). Pathogenic taxa, most notably *Fusobacterium nucleatum* and *Porphyromonas gingivalis*, are central to orchestrating a pro-tumorigenic microenvironment by inducing chronic inflammation, compromising mucosal barriers, and secreting genotoxic metabolites. A paradigm shift in microbiome research has redirected focus from individual species’ pathogenicity to the collective functional evolution of microbial communities (Santacroce et al., 2021). This functional convergence provides a robust scientific foundation for identifying universal biomarkers across diverse populations.

Importantly, the literature on microbiome-based OSCC prediction encompasses four distinct tasks that should not be conflated: (1) detection/discrimination models that distinguish prevalent OSCC from healthy or non-malignant states; (2) malignant transformation models that predict progression from oral potentially malignant disorders (OPMDs) to invasive carcinoma; (3) recurrence/metastasis models that predict post-treatment disease recurrence or nodal metastasis; and (4) prognostic models that predict survival or other long-term clinical outcomes. These tasks differ fundamentally in their biological basis, clinical context, required study design, and validation requirements. Throughout this review, we maintain these distinctions and evaluate the evidence for each category separately.

The high-dimensional and dynamic nature of oral microbiome data offers an unprecedented opportunity to innovate OSCC risk assessment (Han et al., 2025). This advantage facilitates a transition from reliance on conventional clinical factors—such as age, sex, and habits—toward the integration of multidimensional microbial features into predictive models (Wang et al., 2025a; Han et al., 2025; Guerrero-Preston et al., 2017; Huang et al., 2020). Machine learning algorithms, including random forest and extreme gradient boosting (XGBoost), have become cornerstone analytical tools due to their capacity to process high-dimensional data and model complex non-linear relationships (Wang et al., 2026). These diagnostic models have consistently demonstrated superior discriminatory performance and generalizability in independent validation cohorts.

This review systematically delineates the current landscape of OSCC microbiome databases and advancements in risk assessment modeling. We first analyze the molecular mechanisms by which oral dysbiosis mediates oncogenesis and evaluate the reliability of specific microbial signatures as clinical biomarkers. Next, we examine how mainstream oral microbiome databases and OSCC-specific datasets, focusing on the logic and clinical utility of existing risk models. Finally, we assess current bottlenecks regarding model standardization and provide perspectives on leveraging microecological big data to optimize clinical decision-making for OSCC.

## 2 Current status of oral squamous cell carcinoma microbiome databases

### 2.1 Compositional characteristics of the oral microecosystem

#### 2.1.1 Differences between OSCC and healthy oral microbiomes

The healthy oral cavity hosts a dynamic microbial community maintained by salivary flow, oral hygiene, and diet (Van Dijk et al., 2024). At the phylum level, this ecosystem is primarily dominated by *Firmicutes, Actinobacteria, Bacteroidetes, Proteobacteria*, and *Fusobacteria* (Deo and Deshmukh, 2019). Within this community, *Streptococcus* species serve as key primary colonizers essential for biofilm structural stability (Al-Hebshi et al., 2015). In contrast, the OSCC microbiome undergoes profound taxonomic remodeling. While findings regarding microbial alpha diversity are inconsistent—with some studies reporting increased richness due to new niches created by tumor necrosis (Han et al., 2025) and others reporting decreased diversity due to pathogen overgrowth (He et al., 2023). Beta diversity analyses clearly distinguish OSCC from healthy states across saliva, swabs, and tissue samples (Han et al., 2025).

A critical consideration in interpreting these differences is reverse causation: established OSCC lesions can modify local oxygen tension, inflammation, nutritional availability, bleeding, and tissue architecture, thereby altering the microbiome (Wang et al., 2025a). A microbial signature distinguishing an established tumor from healthy mucosa may therefore represent a consequence rather than a cause or early predictor of disease. This distinction has profound implications for biomarker development and model interpretation, as discussed in Section 3.5.

#### 2.1.2 Key genera/species associated with OSCC

Cross-regional meta-analyses have identified stable microbial signatures in OSCC, with the enrichment of genera such as F*usobacterium, Porphyromonas, Prevotella, Peptostreptococcus,* and *Parvimonas* (Crispino et al., 2024). Conversely, commensal taxa such as *Neisseria* and *Corynebacterium* are typically depleted (Kim et al., 2025). Furthermore, non-bacterial kingdoms play active roles in OSCC pathogenesis: *Candida albicans* acts as a key fungal driver through high-capacity acetaldehyde production and *Candida*-bacterial biofilm scaffolding, while high-risk HPV strains interact with mucosal immunity (Wang et al., 2026). However, non-bacterial taxa remain largely uncharacterized in current predictive models.

#### 2.1.3 Alterations in microbial diversity, community structure, and function

OSCC-associated dysbiosis is characterized by reduced community stability and functional shifts. During the progression from oral potentially malignant disorders (OPMDs) to invasive carcinoma, the community undergoes ecological succession from a commensal-dominated to a pathogen-dominated state (Intini et al., 2025). Functional genomics reveals an enrichment of pathways involved in lipopolysaccharide biosynthesis and bacterial chemotaxis, which are closely linked to the development of a pro-tumorigenic microenvironment (Santacroce et al., 2025).

#### 2.1.4 Consistency of *Fusobacterium nucleatum* associations across populations

While *F. nucleatum* enrichment is among the most consistently reported findings in OSCC microbiome studies, the consistency of this association varies across geographic populations, specimen types, and disease stages. Studies from East Asian (Intini et al., 2025; Wang et al., 2026; Crispino et al., 2024), European (Van Dijk et al., 2024; Neuzillet et al., 2021), and North American (Sukmana et al., 2024; Han et al., 2025) cohorts have all reported *Fusobacterium* enrichment in OSCC, suggesting broad cross-population validity. However, the magnitude of enrichment and the specific *Fusobacterium* species or subspecies identified differ across studies, likely reflecting geographic, dietary, and methodological variation (Datorre et al., 2025).

Critically, the direction of association with prognosis appears to depend on disease stage and anatomical subsite. While *F. nucleatum* positivity in OSCC tumors is generally associated with better prognosis in mixed-stage cohorts (Neuzillet et al., 2021; Datorre et al., 2025), it has been identified as a poor prognostic factor in early-stage HPV-negative tongue cancers (Chan et al., 2022). Rather than merely reflecting stage or subsite heterogeneity, this *F. nucleatum* paradox is fundamentally an interkingdom and viral-bacterial phenomenon, where poor prognosis is specific to HPV-negative tongue cancers driven primarily by bacterial-inflammatory pathways. This apparent paradox underscores the need for stratified analyses and careful consideration of clinical context when evaluating biomarker utility.

#### 2.1.5 Relative abundance versus absolute microbial load

A methodological consideration often overlooked in OSCC microbiome studies is the distinction between relative abundance (proportional representation) and absolute microbial load (total bacterial counts). Most 16S rRNA sequencing studies report relative abundance data, which are compositional and subject to the “closed-sum” constraint—an increase in one taxon’s relative abundance necessarily decreases that of others even if absolute counts remain stable. This can generate spurious associations and obscure true biological signals. Future studies using quantitative PCR, flow cytometry, or spike-in controls to measure absolute load in addition to relative abundance would improve biological interpretability (Liu et al., 2025).

### 2.2 Potential mechanisms of the microbiome in OSCC development

#### 2.2.1 Inflammation and immune regulation

Chronic inflammation serves as a critical bridge between microbial infection and tumorigenesis. Specific pathogens such as *P. gingivalis* and *F. nucleatum* persistently activate host immune responses via Toll-like receptors, initiating NF–κB and MAPK signaling pathways that trigger the release of pro-inflammatory cytokines such as IL-1β, IL-6, and TNF-α (Garofalo et al., 2025). This sustained inflammatory milieu promotes tissue damage and aberrant cellular proliferation. Mechanistically, *P. gingivalis* and *F. nucleatum* display active synergy rather than mere co-occurrence; *F. nucleatum* serves as a structural bridge facilitating *P. gingivalis* invasion, which in turn upregulates pro-inflammatory IL-6/STAT3 signaling and matrix metalloproteinases (MMP-2/MMP-9) to accelerate OSCC progression (Song et al., 2025). Direct evidence for these pathways in OSCC tissues comes from immunohistochemical and transcriptomic studies. In addition, microbes facilitate tumor immune escape; for instance, the Fap2 protein of *F. nucleatum* binds to the inhibitory receptor TIGIT on natural killer cells (Wang et al., 2024). This mechanism has been demonstrated in OSCC cell lines and tissue samples.

#### 2.2.2 Metabolites and oncogenic pathways

The oral microbiota influences host physiology through metabolic small molecules. While certain bacteria metabolize ethanol into acetaldehyde, a potent carcinogen that induces DNA adducts (Kashyap and Kullaa, 2024), non-bacterial species—most notably *Candida albicans*—exhibit exceptionally high alcohol dehydrogenase (ADH) activity, acting as predominant acetaldehyde producers that directly contribute to alcohol-related OSCC etiology. In OSCC-related dysbiosis, elevated levels of short-chain fatty acids (e.g., butyrate) may induce oxidative stress and modulate gene expression via histone deacetylase inhibition (Praveen et al., 2024). Salivary metabolomic studies have confirmed altered polyamine and arginine metabolism in OSCC patients, though the direct microbial contribution requires further elucidation (Tang et al., 2023).

#### 2.2.3 Direct interaction between microbes and host cells

Direct physical interactions precisely perturb oncogenic pathways. The FadA adhesin of *F. nucleatum* binds to epithelial E-cadherin, activating β-catenin signaling and upregulating proto-oncogenes such as *c-Myc* and *Cyclin D1* (Wang et al., 2025b). Other species, such as *Capnocytophaga gingivalis*, induce epithelial–mesenchymal transition (EMT), enhancing invasion and metastasis (Song et al., 2025). EMT-related changes in gene expression have been observed in OSCC coculture models. Recent evidence also suggests that *F. nucleatum* inhibits p53-mediated DNA repair, thereby increasing genomic instability (Sukmana et al., 2024). This finding is supported by studies in OSCC-derived cell lines.

#### 2.2.4 Methodological quality and control rigor in mechanistic literature

A critical appraisal of the cited mechanistic studies reveals significant variation in the quality of the evidence. Many *in vitro* coculture experiments lack essential non-pathogenic commensal controls (e.g., assessing *P. gingivalis* or *F. nucleatum* at supraphysiological doses without comparing them to commensal *Streptococcus* species). Furthermore, *in vivo* animal models frequently omit gnotobiotic or antibiotic-cleared baseline controls. Future mechanistic studies should incorporate standardized methodological controls and physiological bacterial densities to distinguish bona fide oncogenic drivers from nonspecific inflammatory responses (Figure 2).

**Figure 2:**
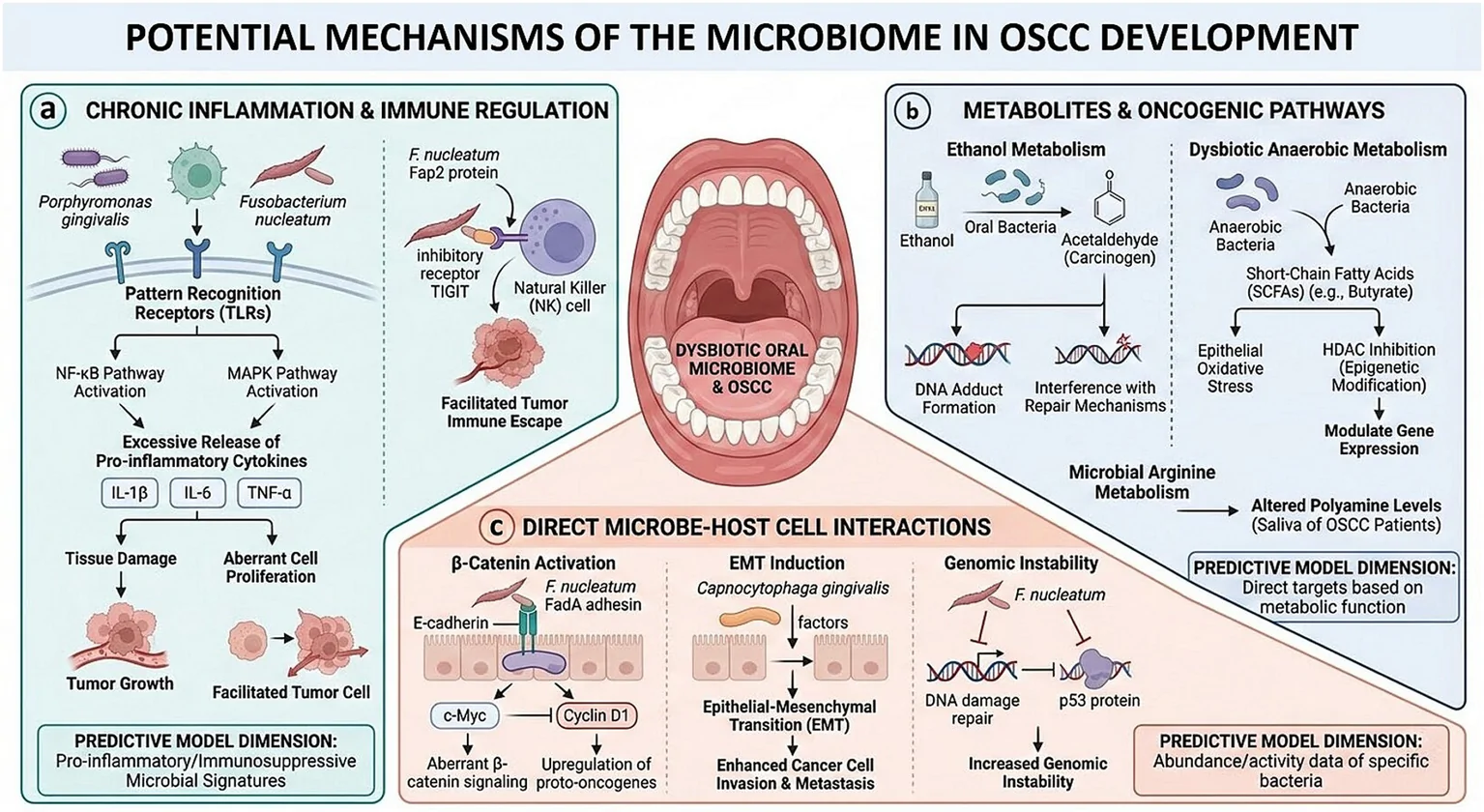
Potential mechanisms and predictive dimensions of the oral microbiome in OSCC development. The diagram illustrates three primary pathways through which dysbiotic oral microbiota contributes to carcinogenesis: **(a)** Chronic Inflammation and Immune Regulation: Activation of TLRs and signaling pathways (NF–κB, MAPK) leading to proinflammatory cytokine release, and immune escape mediated by Fap2–TIGIT interactions. **(b)** Metabolites and oncogenic pathways: Microbial metabolism of ethanol into acetaldehyde, dysbiotic anaerobic metabolism (SCFAs), and arginine metabolism affecting DNA stability and gene expression. **(c)** Direct microbe–host cell interactions: Bacterial adhesion-mediated signaling (e.g., FadA/β-catenin), EMT induction, and interference with DNA repair (p53 inhibition). The shaded boxes indicate the integration of these biological mechanisms into predictive model dimensions, categorizing microbial signatures by inflammatory profiles, metabolic functions, and specific bacterial abundance.

### 2.3 Advantages and potential of oral microbiota as biomarkers

Compared with traditional clinical indicators, the oral microbiome offers unique advantages as a dynamic and non-invasive biological information system (Liu et al., 2025). Saliva collection is non-invasive and requires no specialized clinical procedures, ensuring high patient compliance and broad screening accessibility (Intini et al., 2025). Crucially, microbial disturbances often precede morphological changes detectable by imaging, occurring as early as the OPMD stage (Crispino et al., 2024). This temporal precedence allows microbiome-based models to identify high-risk individuals before irreversible disease progression (Wang et al., 2026).

However, it is essential to distinguish between (a) biological association (microbial differences between OSCC and healthy states) and (b) demonstrated predictive utility (ability to accurately classify or predict outcomes in independent cohorts). While biological associations provide a foundation for biomarker development, they do not, by themselves, establish clinical utility. The evidence for each of these levels is systematically evaluated in Section 3.

### 2.4 Overview of mainstream global microbiome databases

Standardized, high-quality data are foundational for high-precision modeling. The human oral microbiome database (eHOMD) provides authoritative reference sequences and multi-omics data for species identification. (HOMD: human oral microbiome database). Maintained by the ADA Forsyth Institute, it provides detailed 16S rRNA gene reference sequences covering bacteria, archaea, and fungi. Its fourth version integrates high-quality whole-genome sequences, proteomic data, and bioinformatics tools, serving as a standardized reference for species identification and genomic research (ADA Forsyth Institute, 2024). Although eHOMD v4 expands coverage to fungi and archaea, these strata remain underutilized in current predictive models. Additionally, specialized viral repositories such as the Human Oral Virome Database (HOVD) provide essential reference genomes for oral virome profiling yet have not been integrated into OSCC risk algorithms. The Human Microbiome Project (HMP) established a baseline for healthy oral niches (Maki et al., 2021), while the Oral Microbiome Bank of China (OMBC) offers regional specificity for biomarker studies. However, these resources are not specifically tailored for OSCC research (Dohlman et al., 2021). The Cancer Microbiome Atlas (TCMA) provides pan-cancer microbial profiles, but OSCC-specific content remains limited. Table 1 provides a comprehensive comparison of available resources.

**Table 1:** Global microbiome resources and OSCC-specific datasets.

Database / Resource	Target scope	Sample types included	Sequencing modality	OSCC-Specific content / Cohort size	Clinical metadata availability	Accessibility & URL
eHOMD (Human Oral Microbiome Database, 2026; ADA Forsyth Institute, 2024)	Human oral & respiratory tract reference	Saliva, dental plaque, mucosal swabs	16S rRNA, Shotgun WGS (Ref genomes)	Comprehensive taxonomic reference (>770 species); non-cancer specific	High (Genomic, taxonomic, biochemical)	Open access (homd.org)
HMP (Maki et al., 2021)	Healthy multi-site human reference	Saliva, buccal mucosa, tongue dorsum, plaque	16S rRNA, Metagenomic WGS	Baseline healthy controls; no OSCC cases	High (Demographic, healthy baseline)	Open access (hmpdacc.org)
OMBC (Dohlman et al., 2021)	Regional oral microbiome reference	Saliva, subgingival plaque, oral swabs	16S rRNA, Shotgun WGS	Population baseline (East Asian focus); limited OSCC specific subsets	Moderate (Demographics, region, oral health)	Open access / On request
TCMA (Dohlman et al., 2021)	Multi-cancer tissue-resident microbiome	Tissue biopsies, adjacent normal tissue	Shotgun WGS, De-contaminated 16S	Pan-cancer cohort including Head & Neck / OSCC tissue samples	High (TCGA clinical, staging, survival)	Open access (tcma.dfci.harvard.edu)
Cross-Cohort Meta-Analysis Datasets (Wang et al., 2025a; Han et al., 2025; Wang et al., 2026)	Multi-center OSCC research aggregation	Saliva, tissue swabs, surgical biopsies	16S rRNA, Metagenomics, Metatranscriptomics	Integrated cross-regional cohorts (*n* = 500–1,255 samples)	Variable (Staging, habits, age, sex)	Repository-specific (NCBI SRA / GEO)

Despite these valuable resources, a centralized, globally standardized database dedicated exclusively to OSCC—combining longitudinal saliva/swab sampling with standardized clinical, radiomic, and pathological metadata—is currently lacking.

### 2.5 OSCC-specific microbiome datasets

OSCC-specific datasets are increasingly characterized by multi-omics integration (Wang et al., 2025a). While 16S rRNA sequencing remains cost-effective for compositional analysis, metagenomic sequencing provides species-level resolution and functional quantification (Liu et al., 2025). Metatranscriptomics further captures active microbial mRNA, offering insights into real-time host–microbe interactions (Jain et al., 2024). Large-scale meta-analyses, such as a recent integration of 1,255 global samples, are essential to mitigate batch effects and ensure the robustness of identified features (Wang et al., 2025a). Datasets now encompass various sample types, including saliva, lesion swabs, tumor biopsies, and dental plaque (Liu et al., 2025). Different sample types provide complementary information: saliva reflects global oral ecology, while swabs or tissue samples more specifically capture local flora directly associated with the tumor. When building a model, clearly define the sample type underpinning the training data and understanding its ecological implications to ensure clinical applicability.

The sampling site should be treated as a major analytical variable. Saliva, oral rinse, tumor swab, adjacent mucosal swab, and tissue biopsy represent biologically distinct microbial ecosystems and should not be treated as interchangeable inputs. Models trained on one sample type may not generalize to others, and studies combining different sample types without appropriate stratification risk generating non-reproducible findings. This is particularly relevant for clinical application, where sample collection procedures must be standardized for the intended use case (Table 2).

**Table 2:** Comparison of different oral sampling types for microbiome-based OSCC risk assessment.

Sample type	Invasiveness	Advantages	Limitations	Clinical application
Saliva	Non-invasive	Broad surveys, high compliance	Signal dilution	Population screening
Tissue Swab	Minimally invasive	High local resolution	Surface only	High-risk group profiling
Tissue Biopsy	Invasive	Gold standard, mechanistic insights	Sampling bias, procedural risk	Surgical evaluation, research
Oral Rinse	Non-invasive	Convenientl	Diet sensitivity, variable yield	Supplementary screening

The table summarizes the characteristics, data values, and clinical/research applications of common sample types: Saliva: Non-invasive and suitable for broad surveys, though susceptible to signal dilution. Tissue Swab: High diagnostic performance for surface-level microbial profiling in high-risk groups. Tissue Biopsy: The gold standard for in-depth mechanistic studies and surgical evaluation. Oral Rinse: A convenient supplementary tool, though highly influenced by dietary factors.

## 3 Risk assessment models for oral squamous cell carcinoma based on microbiome databases

Risk assessment models underpinned by microbiome databases have demonstrated profound utility across a range of pathological conditions. A growing body of research has established diagnostic and prognostic risk assessment frameworks for OSCC, aiming to meet the clinical needs of early-stage screening and individualized treatment. Model construction facilitates sophisticated analysis of voluminous datasets, empowering investigators to engineer predictive instruments for early oncological diagnosis and precision medicine while providing robust conceptual scaffolding for elucidating the microbiota’s multifaceted role in tumoral identification (Soghli et al., 2000). The methodological landscape has undergone paradigmatic evolution from rudimentary univariate statistical descriptions toward multi-algorithmic parallelism and multimodal data fusion (Wang et al., 2026; Hashimoto et al., 2022).

Throughout this section, we distinguish between models developed and validated within the same cohort (internally validated), models tested on independent datasets (externally validated), and models that have undergone prospective evaluation (prospectively validated). As of 2026, the overwhelming majority of OSCC microbiome models remain at the internally validated stage, with limited external validation and virtually no prospective validation. This evidence gap should guide how we interpret performance claims.

### 3.1 Introduction to disease risk assessment models

#### 3.1.1 Traditional statistical models

In nascent stages of model development, traditional frequentist statistical frameworks—Wilcoxon rank-sum test, chi-square test, Student’s *t*-test—serve as foundational tools for delineating associations between microbial signatures and OSCC susceptibility, instrumental in preliminary filtration of differentially abundant bacterial genera between OSCC and healthy controls (Wang et al., 2026; Kim et al., 2025; He et al., 2024). Regression-based architectures, predominantly logistic regression and Cox proportional hazards models, have been extensively deployed in granular investigation of OSCC and its associated microbiome. Their quintessential merit resides in inherent interpretability, allowing researchers to uncover statistically rigorous correlations between specific bacterial biomarkers and oncogenic phenotypes across diverse cancer typologies (Topçuo, 2021).

#### 3.1.2 Machine learning (ML) algorithms

Machine learning is a major focus in OSCC microbiome research. Dominant methodologies include random forest (RF), support vector machines (SVM), and eXtreme gradient boosting (XGBoost) (Guo and Wang, 2025). By leveraging ensemble strategies, machine learning effectively adjudicates the bias–variance equilibrium, mitigating overfitting risk—a particularly advantageous when navigating high-dimensional, heterogeneous cross-cohort microbiome datasets (Wang et al., 2026). The intricate interplay between the oral microbiomes and OSCC pathogenesis involves complex non-linear interactions. Random forest is uniquely equipped to manage such non-linear feature dependencies, exceptionally well-suited for multifaceted, high-dimensional biomedical datasets (Han et al., 2025). SVM stands as a robust, versatile supervised learning algorithm for both classification and regression, frequently lauded as optimal for training sets with limited sample sizes, effectively circumventing the “curse of dimensionality” and overfitting while maintaining strong generalization (Wang et al., 2021). XGBoost offers unparalleled precision and computational efficiency when processing large-scale cross-cohort datasets, making it ideal for identifying subtle yet critical patterns of microbial dysbiosis.

#### 3.1.3 Deep learning (DL) models

Deep learning handles complex non-linear relationships and extracts meaningful patterns from high-dimensional data, demonstrating outstanding performance in pattern recognition, feature extraction, and predictive modeling. Various deep learning tools have distinct characteristics, though current OSCC applications remain limited. Deep learning can identify novel therapeutic targets, evaluate anticancer drug sensitivity and resistance, develop targeted drugs, improve immunotherapy, monitor tumor recurrence and evolution, discover biomarkers, and predict prognosis and survival (Liao et al., 2023).

Convolutional neural networks (CNNs) convert microbial classification trees into image-like structures to capture complex relationships among microbial communities, proving highly effective for microbial community classification, localization, disease risk prediction, and metagenomics research (Chen et al., 2022; Han et al., 2024; Przymus et al., 2025). In OSCC, CNNs have been applied primarily in radiomics studies rather than microbiome modeling (Yuan et al., 2024). Graph neural networks (GNNs) process graph-structured data, effectively capturing complex relationships between data points and showing great potential in modeling intricate biological interactions (Alharbi et al., 2025). While GNNs have been applied to lung cancer biomarker identification (Zhang et al., 2025a), and generic microbiome classification (Han et al., 2024), OSCC-specific GNN applications have not yet been reported. Autoencoders simplify complex data by reducing dimensionality and highlighting critical features, compressing imaging, clinical, and genomic data into unified latent spaces for combined diagnosis or generating synthetic samples to address insufficient data volume (Przymus et al., 2025; Zhou and Zhao, 2025). In OSCC microbiome research, autoencoder applications remain largely conceptual.

The limited application of deep learning to OSCC microbiome data remains a major research gap rather than an established methodology. Future studies should systematically evaluate whether deep learning approaches offer advantages over classical ML algorithms for OSCC-specific prediction tasks, particularly given the relatively modest sample sizes currently available.

#### 3.1.4 Multimodal data fusion: integrated modeling

OSCC ontogeny and progression are governed by a complex nexus of microbial shifts, host genetics, immune dynamics, and heterogeneous clinical manifestations. Reliance on a single microbiome dimension often encounters confounding variables and limited explanatory depth. The contemporary modeling frontier involves seamlessly integrating microbiome signatures with multidimensional data streams.

##### 3.1.4.1 Metabolomics integration

Zhang et al. (2025c) comprehensively analyzed oral microbiota, tumor transcriptome, and metabolome in OSCC murine models, revealing that saliva from patients with OSCC significantly accelerated tumoral proliferation through synergistic chronic inflammation, cancer-associated fibroblast (CAF) activation, extracellular matrix (ECM) remodeling, and metabolic reprogramming. Saliva-treated cohorts exhibited prototypical cancer metabolism hallmarks. This study represents direct evidence of multimodal integration in OSCC. Significant correlations between specific bacterial taxa and distinct metabolites suggest that microbes influence oral carcinogenesis via metabolic conduits. For instance, *Capnocytophaga* is significantly enriched and negatively correlates with spermine and indole, suggesting a pivotal role in promoting OSCC development (Wei et al., 2025). However, the incremental predictive value of metabolomics data over microbiome data alone has not been systematically evaluated.

##### 3.1.4.2 Clinical feature integration

Li et al. (2023) categorized clinical variables—sex, age, TNM staging, histology, anatomical site—into subgroups, finding that distinct oral microbial taxa (notably *p_Firmicutes, c_Fusobacteriia, o_Fusobacteriales, f_Fusobacteriaceae, g_Fusobacterium*) exhibited differential enrichment, suggesting robust correlation between OSCC clinical parameters and key microbial inhabitants. Granato et al. (2021) observed that *Veillonella* abundance inversely correlated with T stage, implying that higher salivary *Veillonella* may serve as a favorable prognostic indicator. *Granulicatella* exhibits higher prevalence in node-positive patients; concurrent assessment of clinical N stage and *Granulicatella* abundance could evaluate occult lymph node metastasis risk (Eun et al., 2021). Neuzillet’s research found that patients with high intratumoral *Fusobacterium nucleatum* loads often had better prognostic outcomes. Combining clinical staging, *F. nucleatum* quantification, and immunohistochemistry may identify high-risk recurrence populations (Neuzillet et al., 2021). These studies demonstrate feasibility but require prospective validation.

##### 3.1.4.3 Radiomics integration

Recent literature highlights the efficacy of multiview radiomics models integrating clinical data with immunological indices such as the systemic immune-inflammation index (SII) (Meng et al., 2023). Combining SII with optical coherence tomography (OCT) significantly augments diagnostic precision, allowing comprehensive assessment of localized tumoral characteristics and systemic inflammatory states (Yuan et al., 2024). While these studies demonstrate the potential of radiomics in OSCC, integration with microbiome data remains a future direction rather than a demonstrated achievement. Radiomics captures infinitesimal textural variations imperceptible to human eyes. AI models analyzing pixel-level features can predict micrometastatic clusters in lymph nodes (Mp et al., 2025). Conceptually, microbial signatures could provide biological context for radiological findings; fusing radiomic features with microbiome profiles could theoretically yield a panoramic view of tumoral metastatic potential. However, microbiome–radiomics integration has not yet been tested in OSCC cohorts (Figure 3).

**Figure 3:**
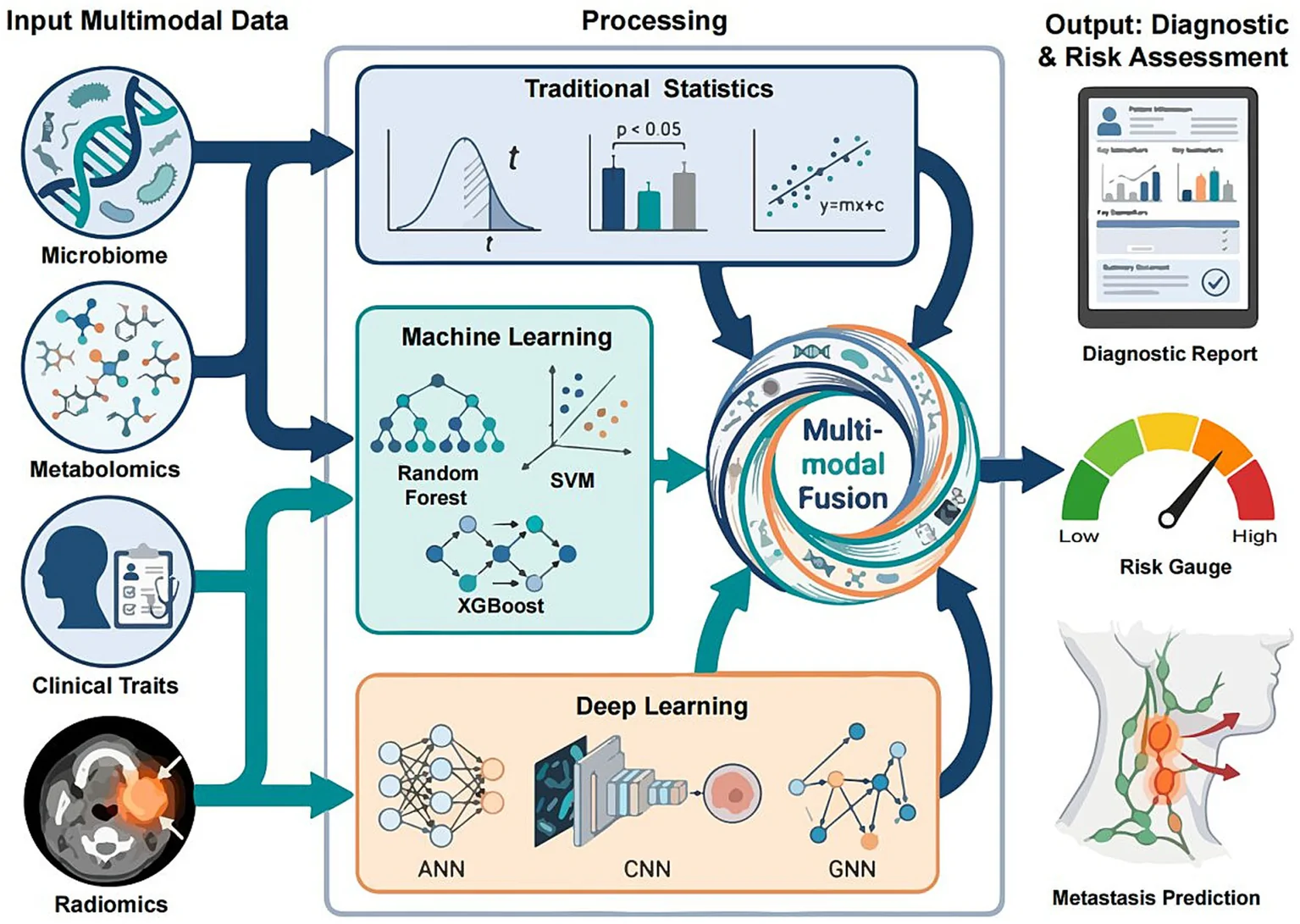
Synthesis of microbiome, metabolome, clinical, and radiomic data—processed through advanced statistical frameworks (Traditional Models, Machine Learning, Deep Learning, and Multimodal Fusion)—enables the construction of high-fidelity OSCC risk assessment models. These integrated tools are ultimately deployed to calibrate or guide the diagnosis and prognostic tracking of OSCC. SVM, support vector machine; ANN, artificial neural network; CNN, convolutional neural network; GNN, graph neural network.

In summary, establishing salivary microbiome models for OSCC necessitates not only 16S rRNA or metagenomic sequencing but also a critical reliance on granular clinical data, including TNM staging, surgical margin status, and longitudinal follow-up. A lack of deep coupling between high-dimensional microbiome data and lower-dimensional clinical, metabolic, or radiomic features leads to a fundamental asymmetry, ultimately resulting in model distortion or failure in recurrence and survival prediction (Table 3).

**Table 3:** Multimodal integration in OSCC microbiome studies: demonstrated vs. conceptual.

Integration type	OSCC studies	Demonstrated performance	Current status
Microbiome + Metabolomics	Zhang et al. (2025c) and Wei et al. (2025)	Improved mechanistic insight	Demonstrated
Microbiome + Clinical Features	Li et al. (2023), Granato et al. (2021), Eun et al. (2021), and Neuzillet et al. (2021)	Incremental predictive value	Demonstrated
Microbiome + Radiomics	None reported	Not evaluated	Conceptual
Microbiome + Transcriptomics	Zhang et al. (2025c)	Improved mechanistic insight	Preliminary
Microbiome + Immunological Indices	Meng et al. (2023) and Yuan et al. (2024)	SII + OCT demonstrated	Early-stage

Summary of multimodal data integration strategies in OSCC microbiome research, comparing demonstrated applications with conceptual frameworks. The integration of microbiome data with metabolomics and clinical features has been demonstrated to enhance mechanistic insights and provide incremental predictive value. Meanwhile, microbiome-transcriptomics and microbiome-immunological index integrations remain in preliminary to early-stage development. In contrast, the joint analysis of microbiome profiles and radiomics remains entirely conceptual, with no published OSCC studies reported to date.

### 3.2 Research progress of microbiome models in OSCC diagnosis and management

#### 3.2.1 Early-stage screening

Timely identification of OSCC and its precursors—oral leukoplakia (OLK) and oral lichen planus (OLP)—is essential for augmenting patient survival. Multiple studies analyzing the microbial architecture of OSCC and OPMDs have identified distinct diagnostic signatures. *Actinobacteria* and *Cyanobacteria* are significantly depleted in OSCC tissues versus adjacent non-malignant tissues (Zhou et al., 2020). Researchers note a reduction in S*treptococcus, Haemophilus, Porphyromonas*, and *Actinomyces*, with concomitant enrichment of Fusobacterium in tumoral sites (Takahashi et al., 2019; Zhang et al., 2020; Zhou et al., 2021). OSCC saliva often exhibits elevated *Peptostreptococcus, Capnocytophaga, Alloprevotella*, and *Fusobacterium*, with diminished *Rothia, Streptococcus*, and *Veillonella*. Transition from oral health to OPMD to OSCC involves a significant *β*-diversity shift, characterized by species richness contraction and selective periopathogen enrichment (Banavar et al., 2021). Current modeling focuses on leveraging salivary microbial traits as a non-invasive screening modality; AI-driven models effectively differentiate OSCC from precancerous lesions, facilitating early intervention. However, screening utility requires demonstration in prospective cohorts—the ability to detect OSCC in asymptomatic individuals before clinical diagnosis has not yet been established.

#### 3.2.2 Malignant transformation prediction models

While several studies have identified microbial shifts during OPMD-to-OSCC transition (Intini et al., 2025; Crispino et al., 2024), dedicated malignant transformation prediction models remain rare. Most studies compare established OSCC with OPMD rather than prospectively following patients with OPMD to identify transformation risk factors. The transition from oral health to OPMD to OSCC involves a significant β-diversity shift (Intini et al., 2025), but longitudinal cohorts have not systematically evaluated the predictive value of baseline microbiome features for future transformation. This represents a major research gap, as OPMD patients constitute the most relevant population for preventive screening and early intervention.

#### 3.2.3 Recurrence monitoring

Postoperative monitoring emphasizes tracking whether the oral microbiota reverts to a “healthy” baseline. The persistence of tumor-associated microbial characteristics postsurgery serves as a potent harbinger of high recurrence risk. Granato’s research reported significant relative abundance increases in *Staphylococcus, Stenotrophomonas, Acinetobacter, Centipeda, Selenomonas*, and *Alloscordovia* in recurrent/metastatic OSCC (Eun et al., 2021). Five microbial signatures—*Leptotrichia trevisanii, Capnocytophaga sputigena, Capnocytophaga, Cardiobacterium,* and *Olsenella*—were identified (Eun et al., 2021) to construct a Random Forest model distinguishing recurrent from primary OSCC with high sensitivity, validating that microbial-based ML models surpassed traditional clinicopathological parameters such as surgical margins and lymph node counts. Another study used XGBoost to develop high-precision recurrence prediction, revealing elevated Eubacterium and Lactobacillus positively correlated with increased recurrence risk, while Staphylococcus and Veillonella correlated with reduced risk. A positive correlation between Eubacterium/Lactobacillus and the PI3K/AKT/mTOR pathway provides a mechanistic cornerstone for future research (Kwack et al., 2025). While these recurrence prediction models show promising performance, most studies are retrospective and internally validated within single cohorts. Prospective monitoring studies are needed to determine whether microbial surveillance can identify impending recurrence before clinical or radiological detection.

#### 3.2.4 Prognostic prediction

Eun et al. (2021) revealed that OSCC with cervical lymph node metastasis exhibited significant enrichment of *Prevotella, Bifidobacterium*, and *Finegoldia*, constructing a random forest model to predict nodal involvement risk. In a contrasting discovery, Neuzillet et al. (2021) found *Fusobacterium nucleatum*(+) OSCC cases exhibited lower recurrence rates, fewer metastatic events, and significantly prolonged overall and metastasis-free survival compared to negative cases. This was corroborated by subsequent studies noting *F. nucleatum* detection in 60–80% of OSCC tumors, often associated with earlier clinical stages and superior disease-specific survival (Banavar et al., 2021; Kwack et al., 2025; Datorre et al., 2025). However, in early-stage HPV(−) tongue cancers, *F. nucleatum* presence is identified as a poor prognostic factor, potentially linked to increased invasiveness and diminished survival (Chan et al., 2022). Such discrepancies likely arise from the inherent heterogeneity of tumor stages and anatomical subsites.

The contradictory prognostic associations of *F. nucleatum* illustrate a critical limitation: biomarker utility may be context-dependent. This underscores the importance of stratified analyses and the need for large, well-annotated cohorts to resolve such discrepancies (Figure 4; Table 4).

**Figure 4:**
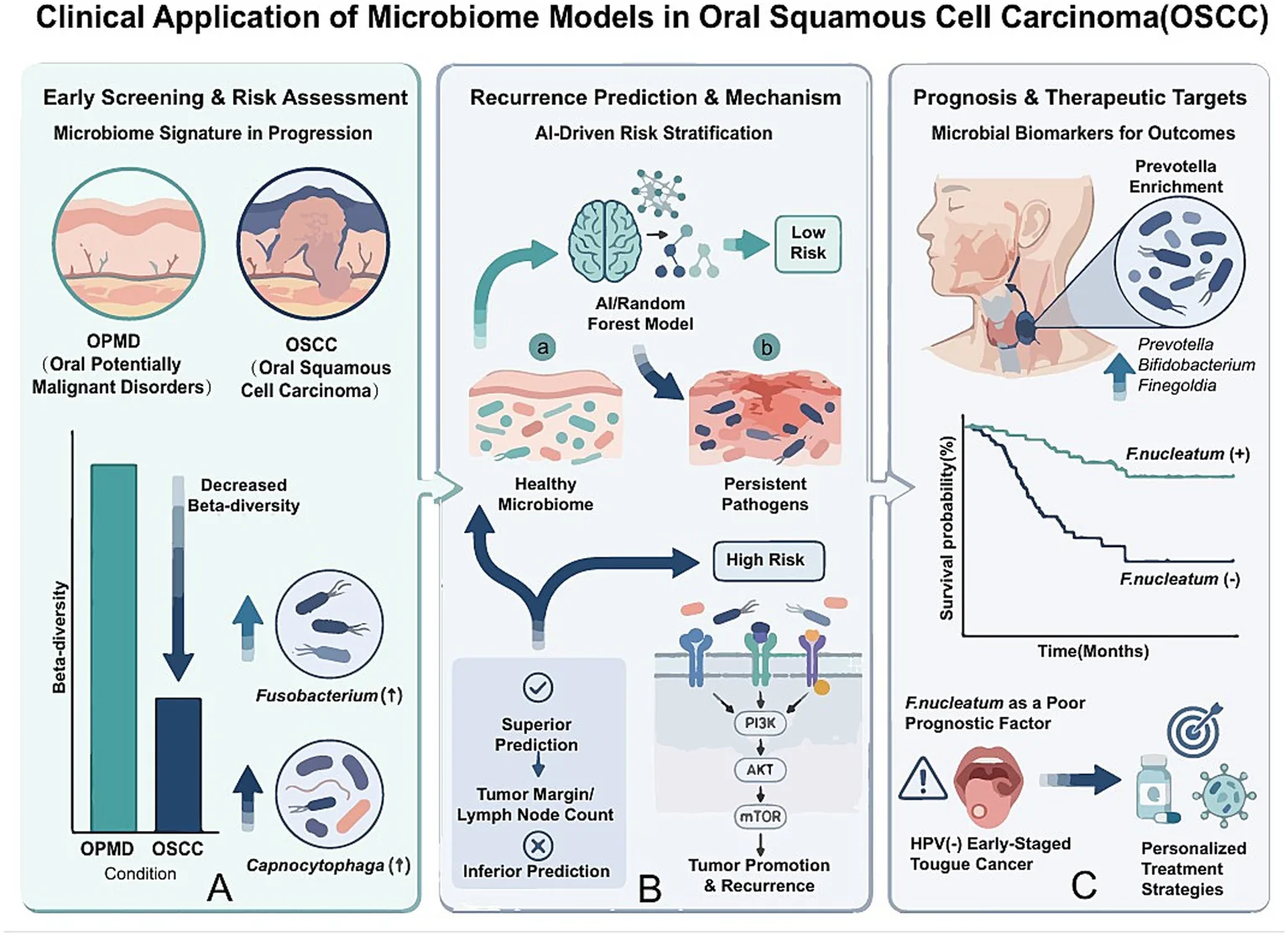
Panel **A** (early screening): Comparison between OSCC and OPMD, showing a reduction in beta-diversity and an upsurge in *Fusobacterium* and *Capnocytophaga* in OSCC saliva. Panel **B** (Recurrence Monitoring): Path-a illustrates a “Healthy Microbiome” (low risk), while Path-b depicts “Persistent Pathogens” (high risk) positively correlated with the PI3K/AKT/mTOR pathway. ML models based on microbial traits show greater reliability than traditional models using margins and nodal counts. Panel **C** (Prognostic Prediction): Displays Prevotella-enriched cervical lymph node icons. Survival curves demonstrate that while *F. nucleatum* (+) patients generally enjoy better survival, the opposite may be true for early-stage HPV(−) tongue cancer.

**Table 4:** Quantitative summary of representative microbiome-based OSCC predictive models.

Study & year	Category / Prediction task	Cohort size & composition	Specimen type & platform	Key feature set / Biomarkers	ML/DL algorithm	Validation strategy	Primary performance metrics (discriminatory & calibration)
Wang et al. (2026)	Cat 1: Diagnostic Discrimination	1,255 multi-cohort samples (OSCC vs. Healthy)	Saliva / Swabs; 16S rRNA	Cross-cohort consensus genus signatures	XGBoost, Random Forest	Cross-cohort & LOCO CV	AUROC 0.88–0.94; Sensitivity 86%, Specificity 89%; LOCO AUROC 0.82
Banavar et al. (2021)	Cat 1 & 2: Screening & OPMD Discrimination	160 subjects (OSCC, OPMD, Healthy)	Saliva; Metatranscriptomics	Active microbial mRNA & host gene transcripts	Random Forest / Gradient Boosting	Independent Holdout Test Set	AUROC 0.92 (OSCC vs. Health), AUROC 0.85 (OSCC vs. OPMD)
Intini et al. (2025)	Cat 2: Malignant Transformation / Progression	95 subjects (OLK, Proliferative Leukoplakia, OSCC)	Saliva; 16S rRNA	*Fusobacterium*, *Porphyromonas*, *β*-diversity shifts	Logistic Regression, Random Forest	10-fold CV	AUROC 0.83 for progression risk stratification
Kwack et al. (2025)	Cat 3: Recurrence Prediction	112 post-op OSCC patients (Recurrent vs. Non-recurrent)	Tissue Swabs & Saliva; 16S rRNA	*Eubacterium*, *Lactobacillus*, PI3K/AKT/mTOR pathway markers	XGBoost, Random Forest	Nested Cross-Validation	AUROC 0.87; Superior to margin/node status alone (AUROC 0.71)
Eun et al. (2021)	Cat 3: Lymph Node Metastasis (cN0 Staging)	88 OSCC patients (Node + vs. Node -)	Tissue Biopsies; 16S rRNA	*Prevotella*, *Bifidobacterium*, *Finegoldia* enrichment	Random Forest, SVM	Leave-One-Out CV	AUROC 0.84 for predicting occult lymph node metastasis
Neuzillet et al. (2021)	Cat 4: Survival & Prognostic Stratification	145 OSCC patients (Longitudinal follow-up)	Intratumoral Tissue; qPCR / 16S	*Fusobacterium nucleatum* load + Immune gene signature	Cox Proportional Hazards, Kaplan–Meier	External Validation Cohort	High *F. nucleatum* correlated with prolonged DSS (HR = 0.42, *p* < 0.01); calibrated C-index 0.78
Desai et al. (2022)	Cat 4: Prognosis in Early-Stage Tongue Cancer	102 HPV(−) tongue cancer patients	Tissue Biopsies; 16S rRNA / qPCR	*F. nucleatum* abundance in HPV(−) early-stage disease	Cox Regression, Random Forest	Internal 5-fold CV	High *F. nucleatum* associated with worse survival in HPV(−) tongue cancer (HR = 2.15)

AUROC, Area Under the Receiver Operating Characteristic Curve; LOCO, Leave-One-Cohort-Out; CV, Cross-Validation; HR, Hazard Ratio; DSS, Disease-Specific Survival.

The table reveals several important patterns: (1) sample sizes remain relatively small, with most studies below 200 participants; (2) external validation beyond the development cohort is rare; (3) clinical comparators are inconsistently reported; (4) calibration and decision-curve analyses are almost entirely absent. These limitations should temper enthusiasm for immediate clinical implementation.

### 3.3 Limitations and challenges

Despite its transformative potential, formidable obstacles remain in the bench-to-bedside transition.

#### 3.3.1 Absence of specialized OSCC microbiome databases

While authoritative repositories such as eHOMD, HMP, and OMBC provide foundational understanding, the lack of a targeted, large-scale database with rich clinical annotations remains a central bottleneck for OSCC clinical translation. Existing baseline databases fail to encapsulate OSCC pathological complexity. HMP primarily delineates healthy cohort microbial baselines; while this helps identify “deviations,” it provides no dynamic map of microecological evolution from premalignancy to terminal metastasis. Although the TCMA pivots toward tumor-associated populations, a unified standardized platform specifically for OSCC salivary microbiota remains absent. The absence of a dedicated OSCC microbiome database with comprehensive clinical annotations is arguably the single greatest barrier to clinical translation.

#### 3.3.2 Confounding variables and noise

A lack of large-scale specialized databases forces OSCC microbiome research into small-sample, single-center studies with poor reproducibility. The oral microbiota is profoundly influenced by geography, ethnicity, and diet. Microbial genera enriched in patients with OSCC from one region may differ significantly from those in another, causing models developed in isolated cohorts to underperform in global or cross-regional validations (Datorre et al., 2025). Many studies fail to sufficiently calibrate for confounding factors such as tobacco, alcohol, periodontal health, and prior antibiotic exposure. Crucially, prior antifungal and antiviral exposures are completely absent from current confounder discussions. Given that antifungal or antiviral treatments significantly alter interkingdom equilibrium and mucosal immunity, their omission introduces substantial unmeasured confounding in the evaluation of microbial biomarkers. The microbial profile of a non-smoking female patient with OSCC differs radically from that of a traditional smoking patient; such cohort biases prevent “universal” models from accurately covering all patient subpopulations (Kim et al., 2025). A lack of standardized sampling—varying from non-stimulated saliva and mouthwash to tissue swabs and biopsies—results in non-overlapping microbial information (Neuzillet et al., 2021; Chen et al., 2020).

##### 3.3.2.1 Reverse causation

As introduced in Section 2.1.1, an established OSCC lesion can modify local oxygen tension, inflammation, nutritional availability, bleeding, and tissue architecture, thereby altering the microbiome. A microbial signature distinguishing an established tumor from healthy mucosa may therefore represent a consequence rather than a cause or early predictor of disease. This is particularly problematic for early screening models, which aim to detect disease before clinical manifestation. Distinguishing causal drivers from secondary colonizers remains a fundamental challenge. Existing studies have adopted various strategies to address confounding, including matching (age, sex, and smoking), covariate adjustment in regression models, stratification by clinical subgroups, and, in limited cases, propensity score approaches. Sensitivity analyses examining the robustness of findings to adjustment for potential confounders are inconsistently reported. Multicenter validation can help identify signature stability across populations, but few studies have achieved this.

#### 3.3.3 Overfitting and generalization capacity

Microbiome data are notoriously high-dimensional, sparse, and compositional—encompassing thousands of microbial species but with sample sizes often in the dozens or hundreds. ML models are highly susceptible to capturing “stochastic noise” specific to particular experimental batches rather than genuine biological signals. Without independent validation sets, models often achieve near-perfect training performance but fail in clinical settings. While leave-one-cohort-out (LOCO) cross-validation bolsters generalization, external validation in diverse clinical environments remains an absolute necessity for real-world application (Wang et al., 2026).

#### 3.3.4 Information leakage

Information leakage is a particularly serious problem in microbiome machine learning. Feature selection, normalization, hyperparameter optimization, and other data-driven preprocessing steps must be performed within the training folds rather than before cross-validation. Using test data to inform any preprocessing step artificially inflates performance estimates. Recommended practices include nested cross-validation, separation of feature selection from test data, patient-level rather than sample-level splitting, and external geographic/cohort validation. Most OSCC microbiome studies do not fully report their information leakage prevention strategies.

#### 3.3.5 Clinical interpretability (the “black box” problem)

Interpretability refers to the capacity to decipher how and why a model generates specific predictions (Desai et al., 2022; Zhang and Fu, 2025). Traditional ML models often operate as “black boxes,” where non-linear transformations across multiple layers obscure decision-making (Zhang et al., 2025b). This lack of transparency poses a significant challenge for clinical adoption (Przymus et al., 2025). A model might identify increased certain bacteria in OSCC but cannot inherently determine whether these are causative agents of carcinogenesis or merely opportunistic colonizers drawn to a tumor-altered microenvironment. To resolve causal ambiguities, “explainable AI” (XAI) techniques such as Local Interpretable Model-agnostic Explanations (LIME) and SHapley Additive exPlanations (SHAP) are increasingly integrated. Integrating XAI allows clinicians to see how each microbial feature contributes to specific patient prognostic scores, aligning computational predictions with biological plausibility (Zhou and Zhao, 2025).

#### 3.3.6 Compositionality, batch effects, and standardization

Microbiome data have specific statistical properties that critically affect model performance. Compositionality (the constant-sum constraint) means that changes in one taxon’s relative abundance necessarily affect others, potentially generating spurious correlations. Sparsity and zero inflation require appropriate statistical methods; ignoring these properties can lead to unstable feature selection. Batch effects and center effects arise from differences in sequencing platforms, protocols, and reagent lots, potentially generating cohort-specific signatures that do not generalize. The choice of normalization/transformation significantly affects downstream results but is rarely justified or compared.

The field currently lacks a globally recognized standard operating procedure (SOP) for sample collection, DNA extraction, library preparation, sequencing, bioinformatics processing, and model development. This heterogeneity limits generalizability and prevents meaningful comparison across studies (Han et al., 2025). Standardization of protocols, coupled with multicenter validation, is essential for clinical translation.

#### 3.3.7 Technical blind spots of 16S profiling and the need for multi-kingdom sequencing

A fundamental technical limitation of existing OSCC models is their near-exclusive reliance on 16S rRNA gene sequencing. Because 16S sequencing is inherently blind to fungal and viral entities, current models capture only a bacteriome-centric window of oral ecology. Transitioning to genuine inter-kingdom characterization requires adopting internal transcribed spacer (ITS) sequencing for mycobiome resolution, shotgun metagenomics for functional and cross-kingdom mapping, and targeted virome enrichment protocols to uncover viral–bacterial driver dynamics.

## 4 Clinical translation prospects and future research directions

Microbiome models are increasingly integrated into precision medicine, offering diagnostic dimensions transcending traditional histopathology. However, current evidence supports promising research findings rather than clinical implementation readiness. Future research must prioritize several key areas. (1) Multicenter, standardized data collaboration: the creation of an integrated OSCC database encompassing metabolomics, signaling pathways, pathomics, radiomics, and granular clinical features is a fundamental prerequisite for clinical translation. (2) Prospective validation studies: the single most important priority for moving the field forward is prospective validation. Internal retrospective validation is insufficient for clinical adoption. (3) Multimodal integration: building upon standardized database infrastructure, multimodal data fusion will serve as a core component of next-generation OSCC diagnostic models. (4) Microbiome-modulating interventions: microbiota reconstruction may eventually be incorporated into clinical protocols. However, these approaches remain experimental; no microbiome-modulating intervention has been validated for OSCC prevention or treatment. Crucially, the therapeutic vision must embrace an interkingdom perspective: bacteriophage therapy itself is an interkingdom biological intervention that uses viral agents to selectively eliminate oncogenic bacteria. Furthermore, future interventions should incorporate antifungal/anti-Candida strategies, e.g., targeting *C. albicans* acetaldehyde production and biofilm scaffolding, alongside HPV-directed immunotherapies to achieve comprehensive microecological risk stratification and treatment (Figure 5).

**Figure 5:**
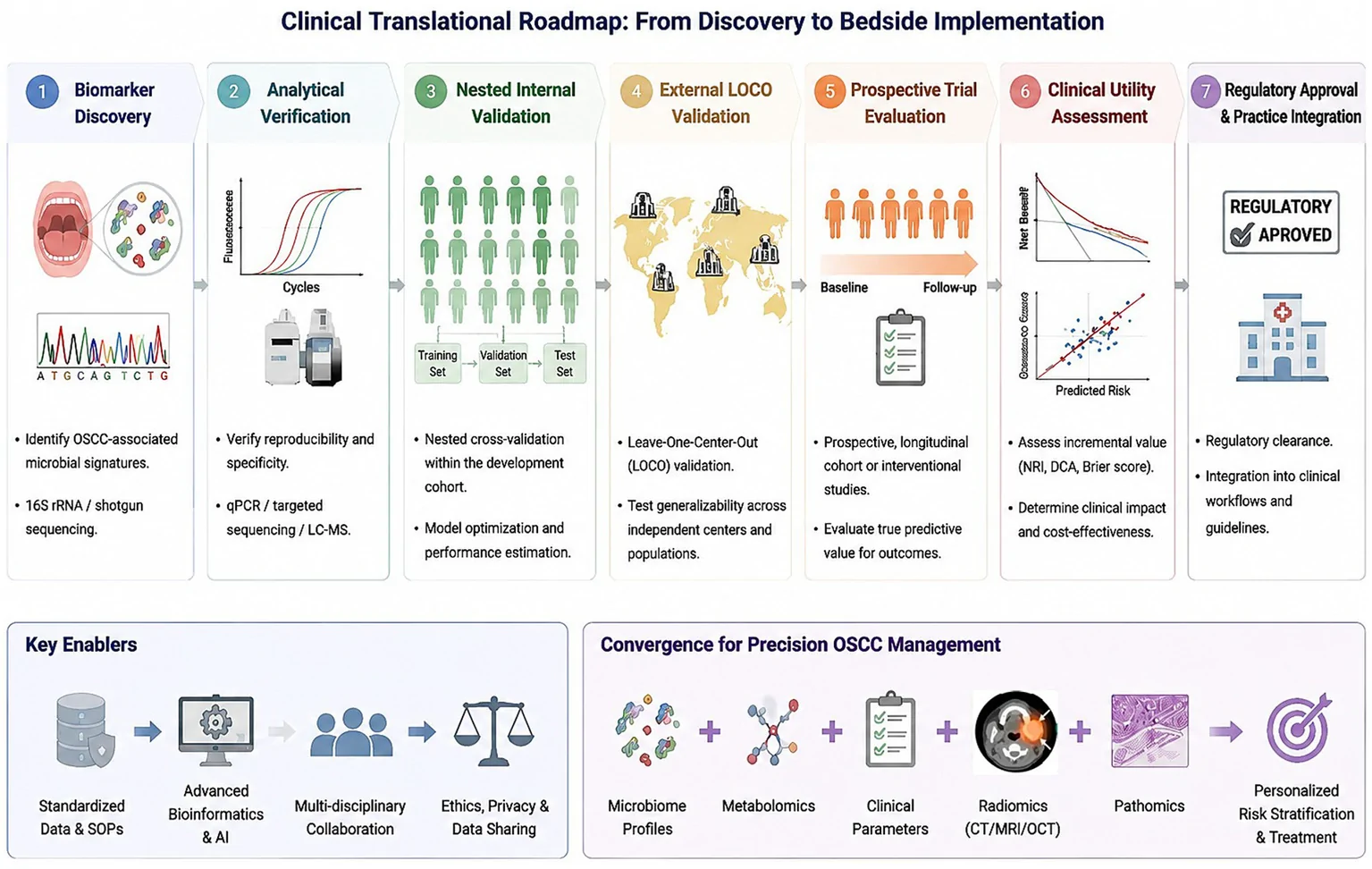
Translating microbiome predictive models into clinical practice. Flowchart illustrating the rigorous seven-stage translational continuum: (1) Discovery and Biomarker Identification → (2) Analytical and Preprocessing Protocols → (3) Internal Nested Cross-Validation → (4) External LOCO Validation → (5) Prospective Longitudinal Cohort Evaluation → (6) Clinical Utility & Decision Curve Analysis → (7) Regulatory Approval & Clinical Deployment.

## 5 Conclusion and perspectives

Microbiome-based predictive modeling represents a promising frontier for non-invasive screening, risk stratification, and the personalized management of OSCC. However, transitioning from statistical association to clinically actionable tools requires rigorous methodological standards. Future research must prioritize: (1) Distinguishing diagnostic discrimination from prospective risk prediction. (2) Adopting strict statistical procedures to address compositionality, zero inflation, and data leakage. (3) Establishing multi-center, standardized OSCC databases accompanied by comprehensive clinical metadata. (4) Integrating multimodal data streams (microbiome, metabolomics, clinical, radiomics) with Explainable AI frameworks to ensure model transparency, calibration, and clinical utility.

Addressing these computational and translational challenges will enable oral microbiome models to deliver precision risk assessments and improve outcomes for patients with OSCC worldwide.

### 5.1 Review methodology

To ensure a rigorous narrative synthesis, literature search protocols were conducted across PubMed/MEDLINE, Web of Science, Scopus, and Google Scholar for English-language publications published between January 2015 and March 2026. The search query incorporated combinations of the MeSH terms and keywords: (“oral squamous cell carcinoma” OR “OSCC” OR “oral cancer”) AND (“microbiome” OR “microbiota” OR “16S rRNA” OR “metagenomics”) AND (“database” OR “machine learning” OR “deep learning” OR “risk model” OR “prediction” OR “classifier”).

Inclusion criteria were: (1) original research or review articles investigating the oral microbiome in OSCC; (2) studies reporting predictive models or diagnostic classifiers using microbial features; (3) studies describing microbiome databases or resources relevant to OSCC; and (4) studies addressing mechanisms linking oral microbiota to OSCC pathogenesis.

Exclusion criteria were: (1) studies not specifically addressing OSCC (i.e., broader head-and-neck cancer without OSCC subgroup analysis); (2) studies without original data or sufficient methodological detail; (3) non-English publications; and (4) conference abstracts without full-text availability. The reference lists of the included articles were manually screened for additional relevant studies. Studies from broader oral cancer or head-and-neck squamous cell carcinoma cohorts were included only if OSCC-specific data were separately reported or when findings were directly relevant to OSCC biology.

## Glossary

AI: artificial intelligence
ANN: artificial neural network
CAF: cancer-associated fibroblast
cN0: clinically node-negative
CNN: convolutional neural network
DL: deep learning
ECM: extracellular matrix
eHOMD: expanded Human Oral Microbiome Database
EMT: epithelial-mesenchymal transition
GLOBOCAN: Global Cancer Statistics
GNN: graph neural network
HMP: Human Microbiome Project
HOMD: Human Oral Microbiome Database
HOVD: Human Oral Virome Database
HPV: human papillomavirus
LIME: local interpretable model-agnostic explanations
LOCO: leave-one-cohort-out
MAPK: mitogen-activated protein kinase
ML: machine learning
NF-κB: nuclear factor kappa-light-chain-enhancer of activated B cells
OCT: optical coherence tomography
OLK: oral leukoplakia
OLP: oral Lichen Planus
OMBC: Oral Microbiome Bank of China
OPMDs: oral potentially malignant disorders
OSCC: oral squamous cell carcinoma
RF: random forest
SCFAs: short-chain fatty acids
SHAP: SHapley Additive exPlanations
SII: systemic immune-inflammation index
SOP: standard operating procedure
SVM: support vector machine
TCMA: The Cancer Microbiome Atlas
TLRs: Toll-like receptors
TNM: tumor, node, metastasis
XAI: explainable artificial intelligence
XGBoost: extreme gradient boosting
